# eB4CAST: An Evidence-Based Tool to Promote Dissemination and Implementation in Community-Based, Public Health Research

**DOI:** 10.3390/ijerph15102142

**Published:** 2018-09-29

**Authors:** Melissa D. Olfert, Rebecca L. Hagedorn, Makenzie L. Barr, Oluremi A. Famodu, Jessica M. Rubino, Jade A. White

**Affiliations:** Division of Animal and Nutritional Sciences, Natural Resources & Design, Davis College of Agriculture, West Virginia University, G016 Agricultural Science Building, Morgantown, WV 26506, USA; rlhagedorn@mix.wvu.edu (R.L.H.); mbarr6@mix.wvu.edu (M.L.B.); OluremiFamodu@tcomn.com (O.A.F.); jrubino@mix.wvu.edu (J.M.R.); jade_white@my.uri.edu (J.A.W.)

**Keywords:** dissemination, implementation, public health, framework, infographic

## Abstract

eB4CAST, evidence-Based forecast C-capture, A-assemble, S-sustain, T-timelessness (eB4CAST), framework was developed from existing dissemination and implementation (D & I) constructs as a dissemination tool to promote community-based program usability and future application in targeted populations. eB4CAST captures and transforms research findings into a dissemination report that shows program need and impact to endorse program continuation and expansion. This is achieved through direct and indirect data collection of community factors and program impact that can showcase the need for program sustainability and potential for future dissemination sites. Testimonials, individual feedback, and program process and outcomes contribute to the direct data while data collected from census, county, and state databases and reports allow for indirect information to be captured and analyzed. Capturing data in the two levels allow eB4CAST to forecast program need and highlight program impact through a footprint. eB4CAST framework for dissemination tool creation is organized into four sections: Capture, Assemble, Sustainability, and Timelessness. Capture encompasses the collection of indirect and direct data related to intervention goals. Assemble is the compilation of the data into a visually appealing and easily understood media. Sustainability encourages the use of dissemination tools to provide forecast of program need and footprint of program impact back to community participants, program leaders, and key stakeholders to endorse program sustainability. Lastly, timelessness encourages cyclic movement through these constructs to continue program monitoring and data sharing to ensure timeless program evaluation and conformation to change in needs. The eB4CAST framework provides a systematic method to capture justification of program need and impact of community-based research that can be modified to fit diverse public health interventions providing a necessary D & I tool.

## 1. Introduction

Dissemination and implementation (D & I) science focuses on understanding the systematic processes of sharing evidence-based interventions and promoting uptake to make sustainable change to health [1,2]. D & I have become a top priority among major funders including the National Institute of Health (NIH) and Centers for Disease Control and Prevention (CDC) due to the substantial gap that remains between research and practice [2]. It is estimated that it takes an average of 17 years for research to translate into community practice and further, only approximately 50% of research ever becomes part of routine practice at all [3]. Therefore, it is crucial that research include a dissemination and implementation component to ensure translation into the community.

Public health is one field where D & I research is important to make widespread improvements on population health across diverse communities. Specifically, within public health research, type 2 (T2) translation, which addresses the progression of evidence-based interventions into sustainable practice and policy change at the population level, is integral to achieving population level health impacts [4]. Research is often done in controlled settings where variables are closely monitored at baseline and post, making it difficult to translate program impact into a real-world setting [5]. To guide T2 translation, there is a call for community involvement to overcome many of the barriers for program adoption [4]. Specifically, community-based research programs often target at-risk populations such as low-income or under-represented individuals who would benefit from continued program dissemination. In order to gain support from both public and private industries, there needs to be standing evidence demonstrating a program’s potential benefit and identify that it has longevity in making a positive public change with measurable impact [6]. Inclusion of Community-Based Participatory Research (CBPR) principles that include community members in disseminating findings and share knowledge gained to all partners is suggested to promote a long-term commitment by all partners [6,7]. Thus, community-based interventions have an increased need to report program need and impact to participants, program leaders, and community stakeholders in order to advocate for program sustainability.

However, to our knowledge, there is no dissemination tool that captures both publicly available and research driven outcome data and transforms it into one visual report developed for community populations. Programs may evaluate publicly available (indirect) data to justify program need when planning an intervention, although it is not always shared with the community at large. Further, the sharing of research outcomes, beyond research presentations and technical reports, is not always guaranteed [8]. Community-based public health researchers can benefit from a tool that allows them to translate scientific findings into visual reports that program leaders can use directly within their communities to tell the story of need for their program and community impact in one visual report. Therefore, the authors have developed eB4CAST as a means to create an infographic story that community leaders can use to share program impact and justify continued implementation.

This manuscript details the development of eB4CAST, a novel dissemination framework, to address the lack of a comprehensive dissemination tool using community-based research to help bridge the current science to practice gap. The evidence-Based forecast C-capture, A-assemble, S-sustain, T-timelessness (eB4CAST) framework addresses the current research to practice gap and seeks to fill guidelines set forth by existing implementation frameworks by disseminating research findings to participants, community members and stakeholders in a format that is accessible to the general population. This framework uses CBPR principles to create a novel dissemination tool through infographic reports that can help bridge the research to practice gap. For future sustainability and change to take place within community-based participatory research, expansion on current dissemination and implementation frameworks is needed. This manuscript will express the rationale behind the presented, novel tool and show a case study of its application in two community-based, public health programs. Currently, this infographic dissemination tool is built by researchers and sent to program leaders for use in community dissemination, with future direction to have reports automated through a web-based platform for sustainability.

### Theoretical Background

Multiple frameworks have been developed to provide guidelines for effective implementation and dissemination of community-based, public health research [9]. The RE-AIM framework [10], the Translation Science to Population (TSci) Impact Framework [4], and the Quality Implementation Framework [11] are three well-known D & I frameworks that provided background and guidance for the development of eB4CAST. eB4CAST seeks to address the guidelines set forth in these frameworks and serves as a much-needed tool to fulfill the functions defined in these frameworks.

Primarily, eB4CAST is an adaptation of the RE-AIM framework which was developed for the health care setting to monitor clinical evidence-based practice [10]. eB4CAST aids in expanding the model to capture the impact of a program in an actual community setting. The original RE-AIM toolkit was developed in order to capture data on programs that can be used to influence policy change looking at health care outcome measures and remains a popularly used framework in NIH funded D & I grants, especially in clinical settings [9,10]. The framework looks at the reach, effectiveness, adoption, implementation, and maintenance to graph the overall health impact from program implementation and was developed in order to document research findings for the data to be translated into current health practice. The RE-AIM framework is limiting in that it is most applicable in a controlled setting and does not account for unknown variables or unforeseen implications of a community setting. By only looking at the study in a well-managed clinical situation it is difficult to determine the unknown variables associated with the program and other unforeseen implications. eB4CAST has been developed to more accurately capture the impact of a program on individuals, program leaders, and communities in real-world environments, while also demonstrating program reach, longevity, and sustainability.

The TSci Impact framework has been developed by the Society for Prevention Research to specifically address Type 2 (T2) research translation [4]. T2 translation research aims to enhance the adoption, implementation, and sustainability of evidence-based or scientifically validated interventions to service systems, such as health care settings, community-based organizations, and schools. The TSci Impact framework looks to a four-step process for evidence-based interventions to have population impact- pre-adoption, adoption, implementation, and sustainability [4]. These four steps are supported by practice-oriented research, practitioner-scientist partnerships, and financing structures. The pre-adoption phase of TSci Impact requires the understanding of factors such as appeal and acceptability of the interventions to its prospective consumers, feasibility of its implementation, and utilizing the key channels which stakeholders use to obtain information regarding evidence-based interventions [4]. Further, for program adoption, an understanding of program impacts is necessary to aid policy makers and implementers in promoting the need for continued community intervention. eB4CAST achieves both of these TSci Impact framework functions through capturing data and assembling it in an easily understood, visual manner to share with program participants, key stakeholders, and policy makers.

Similarly, the Quality Implementation Framework is a four phase, fourteen step process to increase quality implementation [11]. In the QIF, step 13 calls for a supportive feedback mechanism to report intervention results back to community members and stakeholders. This feedback mechanism should provide an effective process to communicate, discuss, and act upon key findings from process and program data. Additionally, this feedback mechanism should allow complex data to be shared with all those involved with the innovation including stakeholders, administrators and frontline practitioners [11]. eB4CAST provides a mechanism in which D & I researchers can complete this step through a visual report to share with community participants and stakeholders for sustainable program impact.

## 2. Methods

### 2.1. Tool Constructs

**Figure 1:**
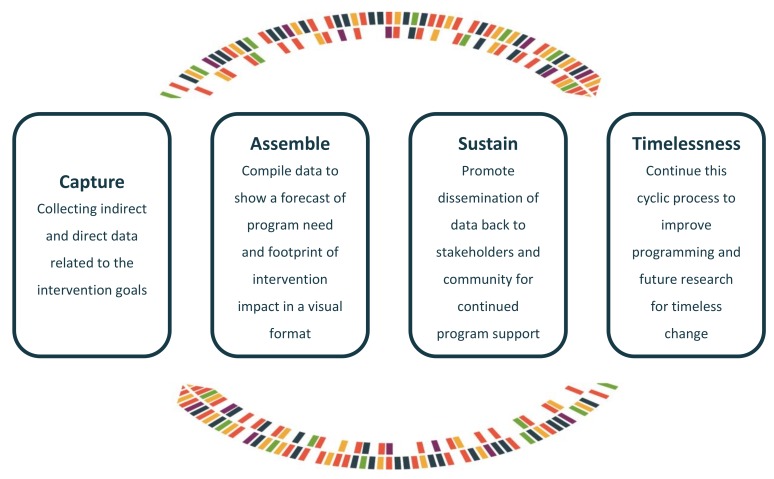
eB4CAST framework constructs.

The forecast and footprint created are combined into a comprehensive report, capturing the pre and post measures of an intervention. This report ensures a program’s suitability and timelessness by providing evidence of its impact to stakeholders in a concise, visual, user-friendly format. eB4CAST describes the need and impact of an intervention using four constructs: capture, assemble, sustain, and timelessness shown in Figure 1.

#### 2.1.1. Capture

Researchers and communities can benefit from using the data from a research driven program to aid community leaders in promoting the need for continued community-based, public health programming. Capturing both indirect and direct data related to program goals provides justification for program implementation and shows the impact the program has had and can continue to have on the community members and address the areas of need. Further, capturing the success of programming in one community can help justify the dissemination and implementation of programming into similar communities in need. Expanding from RE-AIM’s “Reach” construct, which records socioeconomic status and family income but does not account for the culture of the community and incorporating the pre-adoption TSci function, the eB4CAST tool encompasses both the individual and community levels in the “capture” section. The data captured is flexible to the intervention goals and can address the important data specific to each community. Data on recruitment, socioeconomics, and interests of community members captures the individual environment for program implementation with additional community data such as information on staffing of the program, the setting of program, and additional services such as extension and adding information on the intervention environment. Outcome data from process and program evaluations aid in understanding the change that the intervention can have on the community well-being and achievement of program goals. Understanding the entire social setting on an indirect and direct level can help researchers better predict how the program will operate, allowing for forecasting in other potential locations and show program footprint through outcome impacts for program sustainability.

#### 2.1.2. Assemble

The “assemble” section takes complex, research driven data and transforms it into an eye-catching, comprehendible report. There is a divide between scientific and community communication, making it essential for program outcomes to be comprehendible to all populations [12,13]. Further, when using CPBR constructs, sharing data to community members and having their engagement in dissemination is essential for program longevity. Therefore, presenting data from interventions in a manner that can be easily used to share results and program importance without confusion is a promising avenue to promote communication for program sustainability. Additionally, this format is highly acknowledged for its usability in talking to key stakeholders who can aid in vying for community funding. For eB4CAST developed D & I reports, the infographic format is used as a quick, visually appealing avenue to share research findings. Each infographic report is developed to be specific for the program but generally includes the following format. A program overview, community or environment snapshot from the indirect data, program impact from direct data collected, and a take-home message. These reports are sent in both print and electronic versions to the community program leaders to share with their community and stakeholders as in the “sustainability” construct.

#### 2.1.3. Sustainability

It is important to guide the translation of programming from researcher-driven to community-driven for program sustainability. The sustainability construct of eB4CAST promotes the translation of data in a visual format to communities to promote program longevity. The eB4CAST infographic dissemination tool provides communities the power to share their program need and impact with stakeholders and policy-makers as means to endorse continued community-based programming for public health change. This construct of eB4CAST incorporates components of the QIF by promoting a feedback mechanism, the “maintenance” section of RE-AIM to guide incorporation of programming into the community, and the “implementation” and “sustainability” functions of TSci Framework to reach participants and overcome factors influencing implementation to promote supportive policy change. The sustainability construct of eB4CAST empowers community champions to campaign for program longevity and expansion through subjective and objective data (capture construct) of behavior change documented in a comprehensive format (assemble construct) to confirm program effectiveness as a catalyst for continued public health programming.

#### 2.1.4. Timelessness

As communities change, the need of communities will expand, and public health programming will need to be adjusted to fit the community needs. Therefore, D & I frameworks need to be cyclic to ensure program timelessness by continuing to capture data and share impact over time. The “timelessness” section of eB4CAST encourages the promotion of long-term, community level monitoring of implementation outcomes to adjust programming to address everchanging issues in diverse populations. This process allows community-based public health programs to adapt over time and create timeless behavioral and environmental change. The timelessness construct of eB4CAST builds on prior findings to produce a strong understanding of the longevity of the program and adapt as necessary. This cyclic process will allow eB4CAST to continue to monitor community-based programs in longitudinal settings and show impact for years to come.

### 2.2. Tool Data Collection

The eB4CAST tool captures both indirect and direct data to ensure a comprehensive forecast of a particular program environment and to measure the program footprint in order to expand program implementation and dissemination. Indirect data for this project includes data from publicly available sources that is repurposed to showcase the program setting. Direct data are the data being collected from the program, including process and program evaluations, and shows the outcomes from the program. Data collected as indirect and direct data are modifiable depending on the community-based intervention being implemented. For example, a physical activity program’s indirect data may include the physical activity index of the community while a culinary program may include the number of grocery stores in the community. Therefore, the data collected is adjusted to fit the community program being evaluated. The community and environment needs are identified and combined with information of community socioeconomic status, interests, and population statistics. This allows researchers to better identify other locations in need for future dissemination and forecast success in these areas.

The indirect data collected includes county and state statistics that can justify initial program need and be used to gauge program dissemination into similar communities. Information and statistics include, but are not limited to, county and state population, age distribution, ethnicity, gender, unemployment, average family income, average obesity rate, various health behaviors, and food insecurity measures such as percentages of poverty level, families receiving government assistance, and children receiving school breakfast. Community program utilization is also recorded: various programs may include Extension Services, Supplemental Nutrition Assistance Program Education (SNAP-Ed), Expanded Food and Nutrition Education Program (EPNEP), Boys and Girls Club, YMCA, 4-H, Boy Scouts, Girl Scouts, and Health Science and Technology Academy (HSTA). The combination of these statistics creates a comprehensive picture of a current community and areas that need change or support in which a program could be of assistance. The need and feasibility of a project is captured by compiling indirect data from publicly available sources to create a snapshot of the environment in a particular community. This data is assembled into a “forecast” of the need and potential impact of a project. The forecast is a user-friendly, highly accessible infographic that can be distributed to potential stakeholders and participants to gain support for a project.

After a project is implemented, a footprint is created to show the positive impact of a program and used towards ensuring program sustainability and timelessness. The footprint compiles direct measures, both quantitative and qualitative data, collected from the intervention and uses this data to create a second part, the infographic, visually representing the impact of a program in a user-friendly way. The direct data collected from program evaluation tools are sent to the eB4CAST team as part of the “capture” construct. Program measures, including process and program evaluations, are a key part of direct data to show the impact of the community intervention. Additionally, community participants, program leaders, and key stakeholders communicate their experiences and perceptions of different aspects within the program to gain qualitative data beyond typical research measures. Direct participant and stakeholder feedback are vital to forecasting and implementing a program in new areas to advance the progress made and benefits that result.

### 2.3. Tool Infographic Creation

Following data collection, the complex dataset is evaluated by authors and data assembled into a visual infographic and narrative report of the program. The infographic reports are created by the eB4CAST authors using Adobe® Illustrator® software (Adobe Systems Incorporated, San Jose, CA, USA). The infographic reports are reviewed by a graphic designer to ensure aesthetic appeal and data clarity. The infographic report is provided back to the program leaders in both print and electronic format. Program leaders are encouraged to use the infographics to share program outcomes and vie for program dissemination. Thus, the infographic can help “sustain” community programming by showing participants and stakeholders the program’s benefits within the community. This not only helps participants visualize and understand the benefits of the program, but justify the time, efforts, and manpower used for community members and stakeholders to ensure support. Currently, these infographic reports are created by the eB4CAST team but is expected to become and online platform that will ensure a sustainable model for eB4CAST infographics.

## 3. Tool Outcomes

The eB4CAST framework and infographic tool has been used in two community-based programs to date and are presented below as case study examples. This shows the flexibility of eB4CAST to capture data from differing community-based programs and assemble into a dissemination report to promote sustainability of the program for timeless change.

### 3.1. A Childhood Obesity Prevention Program: iCook 4-H

iCook 4-H is an out-of-school obesity prevention program for youth aged 9–10 years and their primary food preparer founded around “Cooking, Eating, and Playing Together” [14,15,16]. This program was tested and disseminated acorss five states: West Virginia, Tennessee, Nebraska, South Dakota, and Maine. eB4CAST infographics were created for iCook 4-H communities in each of the states [17]. Indirect data were chose to align with program outcomes and collected from city-data.com and countyhealthrankings.org. As iCook 4-H is a health-based program, indirect data predominately focused on health determinants [18] and included county level demographics (gender and racial breakdown), population, cost of living, and median household income compared to national average, physical activity, obesity, free lunch and food insecurity percentatges, food index score, and county health rank within the state. This data showcased the justification for program need. Direct data were collected as part of the iCook 4-H project data collection by measures decided upon by iCook 4-H researchers [19] and used secondary for the eB4CAST data. This included process and program evaluations completed by program participants and facilitators. This data captured change from the iCook 4-H program through pre-post evaluation as well as gained feedback on what worked and did not work from the program [19]. Additionally, Ripple Effect Mapping (REM), a qualitative evaluation method in which participants visually map how the program impacted self, peer, and community, was implemented as part of the iCook 4-H program and data was provided for the eB4CAST report [20,21]. This collection of the data represented the “Capture” within the eB4CAST framework and shows the flexibilty to be used within varying programs, as the data was specific to the program at hand. This data was then “Assembled” in the eB4CAST infographic tool to make a visual dissemiantion resource for iCook 4-H that communities can use to promote program continuation and “Sustainability”. The eB4CAST reports can continue to be generated as the program sustains to monitor the “Timelessness” of the program.

**Figure 2:**
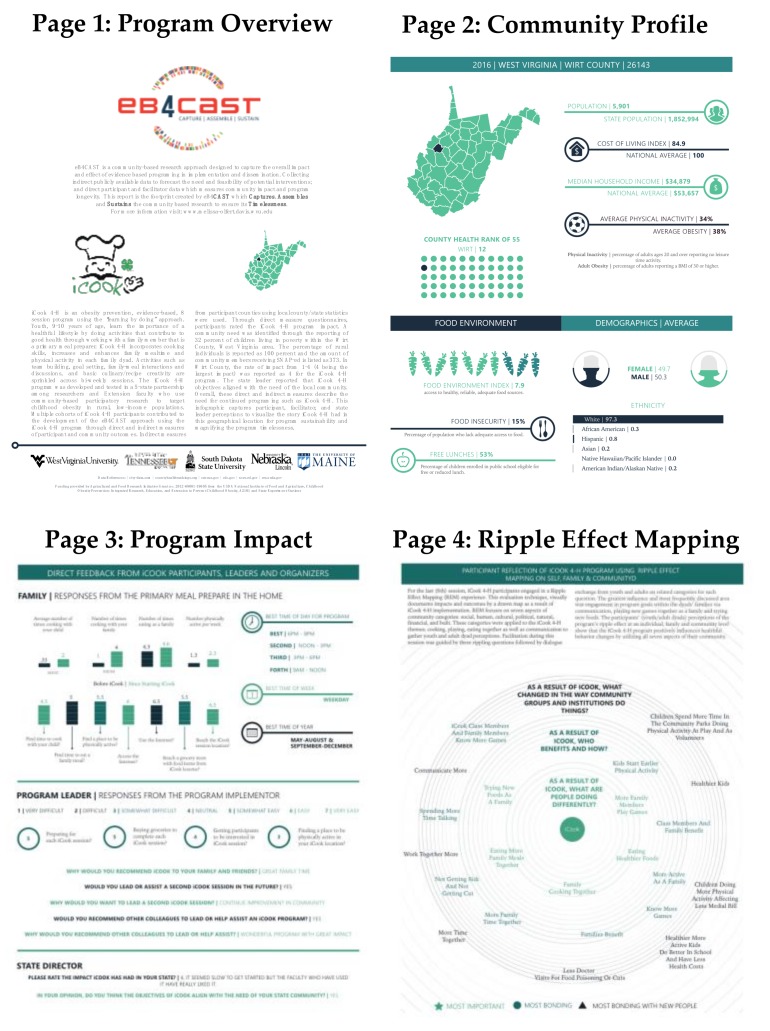
Sample eB4CAST infographic reports from iCook 4-H.

To date, eB4CAST infographic reports have been sent to 14 communities that participated in the iCook 4-H program. The final reports consisted of four pages that included a program overview, community profile (indirect data), program impact (direct data), and Ripple Effect Mapping (direct data). These reports have been evaluated through external review in the iCook 4-H program, with results presented elsewhere [17], but were seen as beneficial to overcome barriers in D & I. As iCook 4-H is now publically available, via the 4-H Mall [22], more infographics can be created as communities purchase and implement the iCook 4-H program and data is provided to researchers for report generation. Sample eB4CAST reports from the iCook 4-H program are shown in Figure 2.

### 3.2. A College Social Marketing and Environmental Change Intervention to Increase Healthy Lifestyles: GetFruved

GetFruved is a research project designed to decrease obesity in older adolescents through behavioral intervention and environmental support on college campuses [23]. This program was originally tested on campuses in four states, West Virginia, Tennessee, South Dakota, and Florida, and has now expanded on over 90 campuses nationwide. Colleges who were enrolled in the randomized control trial (RCT) during GetFruved year 4 were also enrolled to receive an eB4CAST infographic report [24].

Indirect data that aligned for the GetFruved program were collected from both the institution website and national websites and focused on the healthfulness and safety of the environment as well as health determinants [18]. Institutional data included number of residence and dining halls on campus, student enrollment, percentage of students living on campus, gender and race breakdowns, percentage instate residency, and student-to-teacher ratio. National websites were used to collect other campus environment data including campus crime (ope.ed.gov/campussafety/#/), neighborhood crime (neighborhoodscout.com), weather (weatherbase.com), housing and transportation affordability (htaindex.cnt.org/map), walkability (walkscore.com), rurality status (policymap.com/maps), population (census.gov/quickfacts), food environment (ers.usda.gov/data-products/food-environment-atlas/go-to-the-atlas/aspx), and health (countyhealthrankings.org). This data was used create a snapshot of the campus environment and show need for improvement on campus.

Direct data was collected as part of the GetFruved RCT and shared with eB4CAST researchers to create the infographic report. Direct data included the Health Campus Environmental Audit (HCEA) that evaluated campus policy, dining, and recreation [25,26,27,28,29,30,31], the College Environmental Perceptions Survey (CEPS) that determined student and administrator perceptions of the healthfulness of their campus [32], Student and Administrator’s Readiness to Change which evaluated how ready the campus is to make behavioral and environmental change [24], Student and Administrator’s Priorities that examined the top five priorities for change on campus [24], and a Wellness Report card which evaluated campus physical activity, fruit, and vegetable consumption, sleep, and stress compared to national average [23,24]. Following the intervention, pre and post data were shown on the eB4CAST report for all scales to show change made through the GetFruved intervention.

**Figure 3:**
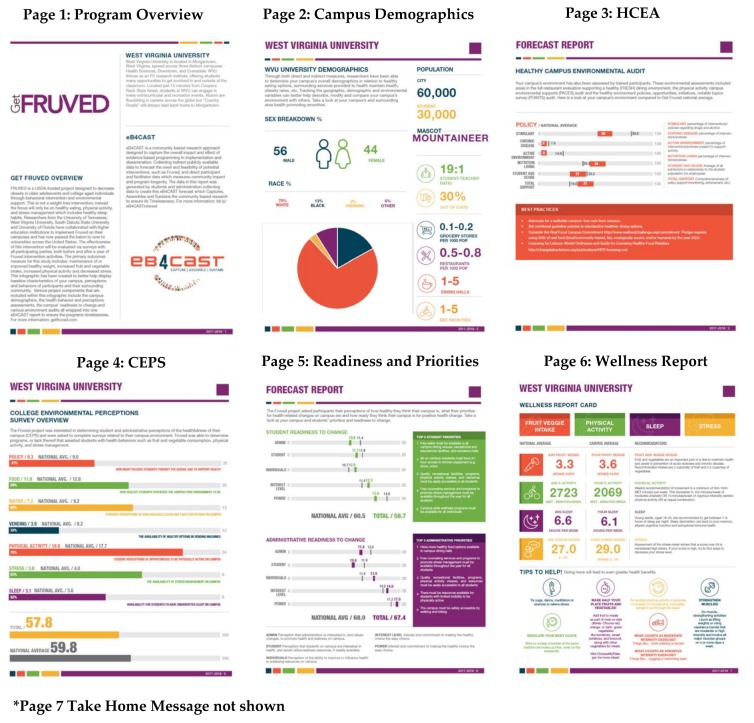
Sample eB4CAST infographic reports from GetFruved.

To date, 66 college campuses have received eB4CAST infographic tools to use to show the impact of GetFruved on campus and sustain the program. Depending the data provided, the final reports consisted of six to eight pages and were provided to campuses after collection of baseline data and post intervention data to show allow campuses to monitor their own change. These reports were evaluated, similar to the iCook 4-H study, with results submitted elsewhere [33]. The GetFruved toolkit will be publicly available after the completion of the intervention and eB4CAST reports can be made for each campus to promote dissemination. Sample eB4CAST infographics from GetFruved are shown in Figure 3.

## 4. Discussion

D & I research has faced many barriers to successful translation for public health change [9]. This manuscript describes the development and theoretical basis of the eB4CAST framework and showcases two case studies of the eB4CAST tool in use for community-based, public health interventions. eB4CAST framework combines the concepts of capture, assemble, sustain, and timelessness into an evidenced-based dissemination tool. The infographic D & I tool uses direct and indirect data to create a visual media that highlights program need and impact on community participants. By incorporating components of previous D & I theoretical frameworks [4,10,11], eB4CAST aims to overcome barriers of successful D & I and provide researchers a means to promote T2 translation for population health improvement. In order to translate research into the public health sector to create population-based change, it is essential to develop a tool for measuring the long-term reach and effectiveness of community programs. Using the proposed eB4CAST model can improve the dissemination and implementation of community-based research to ensure longevity and timelessness.

Although D & I and CBPR principles call for sharing results with community partners, there is evidence that traditional methods of research communication are ineffective for community dissemination. Often researchers are often not incentivized to spend time developing dissemination materials for nonscientific audiences and instead are encouraged to focus on developing manuscripts to disseminate findings in academic journals [8]. Because of these priorities and pressures from employers, few evidence-based tools exist to help researchers effectively communicate their work to community partners [34]. As a result, many researchers may struggle to make their research accessible to and easily understood by stakeholders and community members. eB4CAST infographics provide a means to take complex data and share with communities in an easily understood way, overcoming one of the challenges of dissemination [35,36,37]. Infographics have shown to be useful as educational materials and in aiding the understanding of research [38,39].

Further, eB4CAST infographics can also support communication with funders and stakeholders. When speaking with funding agents, such as policy makers, evidence needs to be presented efficiently to provide a clear impact as time communicating is often limited [40]. Using visually appealing infographics has been one recommendation for communication with policy makers as a means to grab attention and make the programs message known. Infographics are advantageous when communicating quickly as they leverage visual compacity, which is the brain’s most dominant compacity, making communication quicker than text alone [37,40]. This allows programs seeking funding to quickly share their message in the limited time available. The eB4CAST infographics allow programs to vie for funding using visual cues to show program impact and further need in the community. The goal of eB4CAST is to have an effective standard tool to measure effectiveness during implementation, but also to create a model for the future value of the program. In order to have an impact on public policy it is essential to accurately document a program’s long-term duration through a proven method. In order to have an impact on public policy it is essential to document a program’s long-term duration through a proven method. eB4CAST is designed to be suitable for a wide range of community-based interventions and has being implemented for two CBPR projects. This highlights the flexibility of eB4CAST to fit within diverse populations with varying research outcomes. Therefore, eB4CAST provides a systematic method to capture the future value of the program in new untapped communities. It is suggested that the community-based, public health interventions moving forward adapt the eB4CAST framework to aid researchers in D & I of programming. However, this approach may also require a higher level of commitment and resources, which some organizations may be unable to provide [8]. It is suggested that moving forward, eB4CAST will function as an online, interactive infographic platform, eliminating the time and monetary resources necessary to create dissemination reports.

As with any new framework, eB4CAST is not without limitation. First, the eB4CAST framework relies on the capture and assemblage of constructs to be accomplished for the development of the eB4CAST infographic to accomplish the additional constructs. Although this is a cyclic process over time to continue program monitoring and necessity, the constructs must be achieved linearly. Second, the eB4CAST infographic tool is currently created by researchers, which requires a large resource allocation to achieve completed infographics. Additionally, this requires community programs to provide data back to researchers for infographic creation prior to continuing with program dissemination and the sustainability construct. Therefore, it is envisioned that eB4CAST infographics will be transformed into a web-based platform in which community members can directly input their data and received a generated report. This makes the eB4CAST model more sustainable. Lastly, the eB4CAST framework has not been tested vastly. To date, only two community-based programs have used this framework and dissemination tool. Although both programs had positive feedback from community participants and stakeholders, the long-term impact of using the eB4CAST infographics is not known at this point. Additional research will be necessary to evaluate the timelessness of the programs that implemented the eB4CAST framework. Other community-based programs are also encouraged to collaborate with eB4CAST researchers to use this framework and test the model in other public health interventions and beyond.

## 5. Conclusions

The eB4CAST framework supports community-based, public health intervention in translating research into the community through an infographic dissemination tool. This tool can be used to disseminate program impact back into communities following community-based participatory research interventions and overcome previous barriers of D & I. eB4CAST is available for use and testing in other CBPR and public health interventions and the authors encourage use of the tool to aid in dissemination and implementation.
